# Subtype Classification, Immune Infiltration, and Prognosis Analysis of Lung Adenocarcinoma Based on Pyroptosis-Related Genes

**DOI:** 10.1155/2022/1371315

**Published:** 2022-10-12

**Authors:** Qi Liu, Liguang Fang, Chundi Gao, Cun Liu, Haiyang Yu, Jibiao Wu, Lin Hou, Changgang Sun

**Affiliations:** ^1^College of First Clinical Medicine, Shandong University of Traditional Chinese Medicine, Jinan, 250014 Shandong, China; ^2^College of Chinese Medicine, Weifang Medical University, Weifang, 261000 Shandong, China; ^3^State Key Laboratory of Component-based Chinese Medicine, Tianjin University of Traditional Chinese Medicine, Tianjin 301617, China; ^4^College of Chinese Medicine, Shandong University of Traditional Chinese Medicine, Jinan, 250014 Shandong, China; ^5^Qingdao Academy of Chinese Medical Sciences, Shandong University of Traditional Chinese Medicine, Qingdao, 266112 Shandong, China; ^6^Department of Oncology, Weifang Traditional Chinese Hospital, Weifang, 261041 Shandong, China

## Abstract

The effect of pyroptosis-related genes (PRGs) on the tumor microenvironment (TME) in lung adenocarcinoma (LUAD) remains unclear. Thus, this study is aimed at evaluating the prognostic value of PRGs in patients with LUAD and to elucidate their role in the TME and their effect on immunotherapy. Transcriptomic and clinical data were obtained from the Cancer Genome Atlas and the Gene Expression Omnibus databases (GSE3141, GSE31210). Patients with LUAD were classified using consistent clustering, and the differences in the TME for each type were determined using the ESTIMATE and CIBERSORT algorithms. PRGs were screened using univariate regression analysis, and a prognostic risk model was constructed using LASSO regression analysis. The tumor mutational burden and the tumor immune dysfunction and exclusion algorithms were used to predict therapeutic sensitivity in LUAD patients. Then, we evaluated the potential therapeutic interventions using the GDSC database. LUAD patients in cluster 2 had significantly shorter overall survival and progression-free survival rates, lower immune scores, and higher infiltration of T follicular helper cells than those in cluster 1. We used five PRGs to classify patients with LUAD into different risks groups and found that the high-risk group is sensitive to immunotherapy; however, its immune-related pathways were inhibited, which may be related to tumor metabolic reprogramming. Lastly, we identified several potential therapeutic drugs for application in low-risk patients who were less sensitive to immunotherapy. Overall, our findings showed that PRGs can be used to predict prognosis and may aid in the development of personalized therapeutic strategies in LUAD patients.

## 1. Introduction

The incidence of lung adenocarcinoma (LUAD) continues to increase, and surgical resection, chemotherapy, and radiotherapy remain the primary methods of clinical intervention in this disease [1]. Recent reports have highlighted the potential of immunotherapies in lung cancer, specifically immune checkpoint inhibitors; however its application is not always warranted [2]. Tumor immunotherapy primarily works via the activation of the host immune cells which enhances the body's natural antitumor immune response allowing for the specific eradication of minimal residual tumor lesions and the targeted inhibition of tumor growth [3]. Targeted immune checkpoint inhibitors provide a suitable treatment strategy for patients with advanced LUAD and greatly improve their prognosis [4]. In addition, these immunotherapies can be combined with other interventions to improve clinical outcomes depending on the stage of the disease and the condition of the patients. However, different LUAD patients present with different immunotherapy effects [5]. These differences may be related to differences in the tumor microenvironment (TME) making it necessary to determine the degree of immuno-sensitivity in these patients.

Pyroptosis is a form of immunological cell death (ICD) strictly regulated by inflammatory factors which can be divided into both classical and noncanonical pathways [6]. Classical pyroptosis is mediated by inflammasomes, such as NOD-like receptor protein 3 (NLRP3), which directly activates caspase-1, cleaves gasdermin D (GSDMD) to form pore membranes and induces downstream proinflammatory cytokine maturation and release of pro-IL-1*β* and pro-IL-18 [7]. Noncanonical pyroptosis is triggered by caspase-4/5/11 which cleaves GSDMD to form -NT fragments which promote localized cell death [8]. Finally, pyroptosis causes cells to swell, rupture, and release their intracellular inflammatory substances which then trigger an inflammatory response [9]. Dead tumor cells release ATP activating the NLRP3 inflammasomes in dendritic cells, stimulate CD8+ T cells to produce interferon-*γ* (IFN-*γ*), and enhance IL-1*β*-dependent antitumor immunity [10]. Pyroptosis inhibits tumor proliferation and metastasis by altering the permeability of cell membranes, promoting tumor cell lysis and death, and releasing cellular contents. GSDMD enhances the cytotoxic T lymphocyte killing effect in LUAD [10]. Wang et al. suggest that the application of a bio-orthogonal splicing system targeting the gasdermin protein could activate apoptosis, reshaping the tumor immune microenvironment, activating T cell-mediated antitumor immune responses, and facilitating a strong antitumor effect [11]. Pyroptosis mediates the function of various immune cells and regulates the TME [12]. Currently, immune checkpoint inhibitors (ICIs) have been shown to be effective in LUAD [13]. However, the underlying mechanisms resulting in reduced efficacy in some cases of LUAD remain unknown. Despite this, we believe that the application of pyroptosis inducing therapies may help to overcome the limitations of ICIs. Therefore, we further evaluate the effect of pyroptosis on TME in patients with LUAD based on the relevant methods of bioinformatics [14–16].

In this study, we integrated data from TCGA and GEO databases to comprehensively analyze the effect of pyroptosis on the TME in LUAD; interpret the impact of the TME on the survival of patients with LUAD; and provide ideas for investigating the mechanisms involved. Additionally, a new model was developed to predict the prognosis of LUAD patients, screen immunotherapy-sensitive patients, and provide individualized treatment plans. This study demonstrates that pyroptosis acts on the TME of LUAD and affects patient prognosis and treatment.

## 2. Materials and Methods

### 2.1. Data Source

We downloaded the transcriptome and clinical data of 54 healthy patients and 497 LUAD patients from the TCGA database (https://tcga-data.nci.nih.gov/tcga/). We then combined this with the available clinical data to produce a master data set comprising of 468 patients with complete survival time and survival status and then divided these into training and validation sets. The training set was used to construct the model, which was then validated using the validation set. Microarray data were downloaded from the GEO database (GSE3141, GSE31210) (https://www.ncbi.nlm.nih.gov/geo/) and batch correction was performed before this data was used to further validate our novel prognostic model.

### 2.2. Acquisition and Analysis of Pyroptosis-Related Genes (PRGs)

Based on previous studies, 43 PRGs were identified [17–20] (Table S1). Differentially expressed genes (DEGs) were identified using the “limma” software package and depicted using heat maps. A network diagram was constructed through correlation analysis.

### 2.3. Subtypes of LUAD Defined by Pyroptosis

A consensus clustering algorithm was used to identify optimal subtypes based on a matrix composed of PRGs. We classified LUAD subgroups according to the expression of PRGs. Increase the clustering variable (*K*) from 2 to 9, and further find the appropriate *K* value to determine the appropriate subtypes. The above analysis is performed in the R package “ConsensusClusterPlus” The classification was repeated 1000 times to ensure stability [21]. Further, principal component analysis (PCA) was performed to prove that cluster1 and cluster2 can be divided into two groups.

### 2.4. Immunocorrelation Analysis of the Two Subtypes

The ESTIMATE, stromal, and immune scores were assessed for LUAD samples using the ESTIMATE algorithm [22] (Table S2). Further, the proportion of 22 immune cell types in LUAD was assessed using the Cell Type Identification by Estimating Relative Subsets of RNA Transcripts samples (CIBERSORT) algorithm to determine cell types (Table S3). The random sampling algorithm consists of 1000 permutations. Only CIBERSORT (*P* < 0.05) were included.

### 2.5. Gene Set Enrichment Analysis

We further analyzed the biological differences among different risk groups through GSEA and NES (*P* < 0.05) and FDR (*q* < 0.25) were used to identify significantly enriched gene sets. The GSEA4.1.0 tool was used to detect the KEGG pathways.

### 2.6. Construction of a Prognostic Risk Model

We performed univariate regression analysis and calculated the *P* value. When *P* < 0.5, it was included in further analysis to construct a LASSO regression model. The R language package “glemnet” returns a series of lambdas (*λ*s) values and risk models. Further, the “cv.glmnet” function in the “glmnet” package was used to perform ten-fold cross-validation, and the *λ* value with the smallest average cross-validation error was selected, and the LASSO model corresponding to this value was the ideal prognostic risk model constructed. Finally, five best candidate PRGs for building risk prediction models were screened out (BAK1, CYCS, NLRC4, NLRP1, and NOD1). Risk score was calculated according to the following formula: RiskScore = (exprgene1 × Coefgene1) + (exprgene2 × Coefgene2) + ⋯+(exprgene n × Coefgene n) (Table S4). Based on the median risk score, the patient sample was divided into high-risk and low-risk groups. The Kaplan-Meier curve and ROC curve were used to further evaluate the prognostic value of the prognostic risk model. We performed univariate and multivariate analyses of clinicopathological characteristics and risk scores, confirming that the model can act as an independent predictor of clinical outcome. In addition, in order to verify the clinical application value of the constructed model in LUAD, the relationship between the model and clinicopathological characteristics was analyzed using chi-square test.

### 2.7. Immunotherapy Effect Analysis of the Prognostic Risk Models

Tumor mutation load in patients was calculated using the R package, “maftools” [23], and the likelihood of an immunotherapy response was predicted using the TIDE algorithm [24].

### 2.8. GDSC Database

Drug sensitivity data were downloaded from the GDSC website (http://www.cancerrxgene.org/). The IC50 values of compounds obtained from the GDSC website were predicted using the R package, “pRophetic”.

### 2.9. Statistical Analysis

Statistical analysis was done using R (version 4.0.3). The Wilcoxon test is used to extract the PRGs difference genes between normal samples and LUAD samples. The Kaplan-Meier method performed survival analysis and a log-rank test was used to determine the significance of the difference. The distribution of clinical pathologic features between the two groups, the classification variable was chi-square tested, and the continuous variable was the Student's *t*-test. The Mann–Whitney test was used to compare immune cell infiltration and immune-related pathways between the two groups. Correlation between high- and low-risk groups identified using spearman correlation analysis and the infiltration of immune cells. *P* < 0.05 is statistically significant for the difference.

## 3. Results

### 3.1. Upregulation of PRGs Expression in LUAD Samples

**Figure 1:**
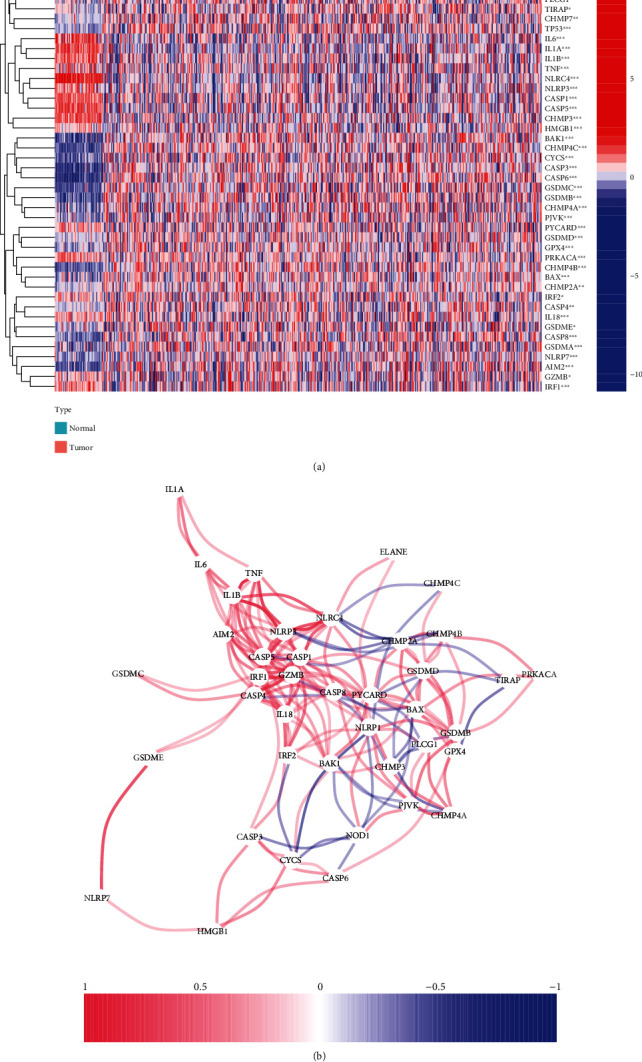
Expression of PRGs in LUAD samples. (a) Heat maps were used to show the differential expression of PRGs. Blue represents downregulated PRGs, red represents upregulated PRGs. (b) The interaction network of PRGs, negative correlation in blue, positive correlation in red.

Significant differences were found in the expression levels of PRGs between LUAD and normal samples (Figure 1(a)) (*P* < 0.05). Further, 26 risk genes (ELANE, GZMB, CHMP7, TIRAP, CHMP2A, PLCG1, GSDMD, CASP4, BAX, GPX4, CHMP4A, CASP8, CHMP4B, TP53, GSDME, PJVK, CYCS, CASP3, BAK1, CASP6, GSDMA, CHMP4C, GSDMB, NLRP7, GSDMC, and AIM2) (log FC>0) and 17 safety genes (IL6, NLRC4, CASP5, IL1A, IL1B, CASP1, NLRP3, IRF1, CHMP3, NLRP1, PYCARD, IL18, PRKACA, TNF, NOD1, HMGB1, and IRF2) (log FC< 0) were identified (Table S5). The interaction of PRGs was further investigated by constructing a correlation network (cutoff = 0.18) (Figure 1(b)), which revealed that pyroptosis may regulate the development of LUAD.

### 3.2. The Survival Status, Immune Score, and Immune Cell Infiltration in Different Subtypes of LUAD Samples

**Figure 2:**
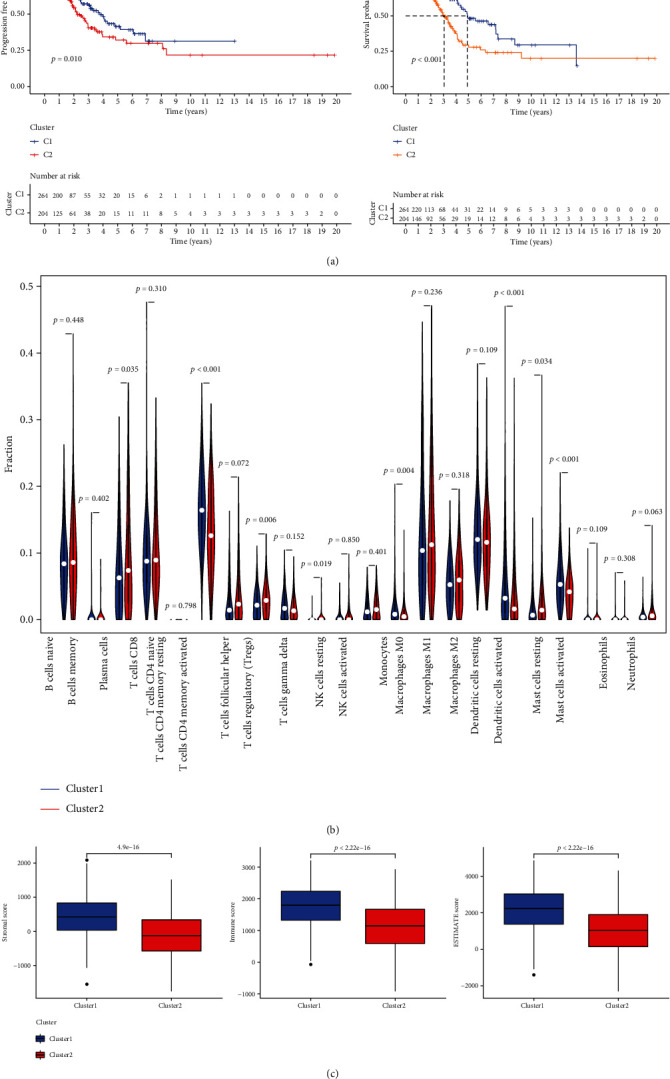
The clinical features, survival status, immune score, and immune cell infiltration of different subtypes of LUAD. (a) OS and PFS curves of two groups of LUAD patients were obtained from TCGA database. (b) There were differences in immune cell infiltration between the two subtypes. (c) Immune score, ESTIMATE score, and stromal score of subtypes in both groups.

We found that when *K* = 2, based on 43 PRGs, 468 LUAD samples were divided into appropriate clusters (cluster 1 and cluster 2), with low inter-group correlation and highest intra-group correlation (Figure S1). PCA analysis further demonstrated that patients can be well divided into two subtypes (Figure S2). PFS (*P* = 0.01) and OS (*P* < 0.001) were shorter in patients with LUAD in cluster 2 than those in cluster 1 (Figure 2(a)). Herein, the relationship between the clinical features and the different subtypes of LUAD was also discussed. Our results suggest that the subtype defined according to PRGs expression is closely associated with the heterogeneity of patients with LUAD. Furthermore, the ESTIMATE score, immune score, and stromal score (Figure 2(c)) of LUAD patients in cluster 1 were found to be higher than those of patients in cluster 2. Compared with that in cluster 2, higher proportion of resting mast cells, monocytes, and T cells and resting dendritic cells was found in cluster 1, while higher proportion of plasma cells, T cells follicular helper infiltration, and dendritic cells was found in cluster 2 (Figure 2(b) and S3). These results suggest that the aggregation subsets based on PRGs are closely related to the immune microenvironment.

### 3.3. Construction of the Prognostic Risk Models Based on TCGA Training Cohort

**Figure 3:**
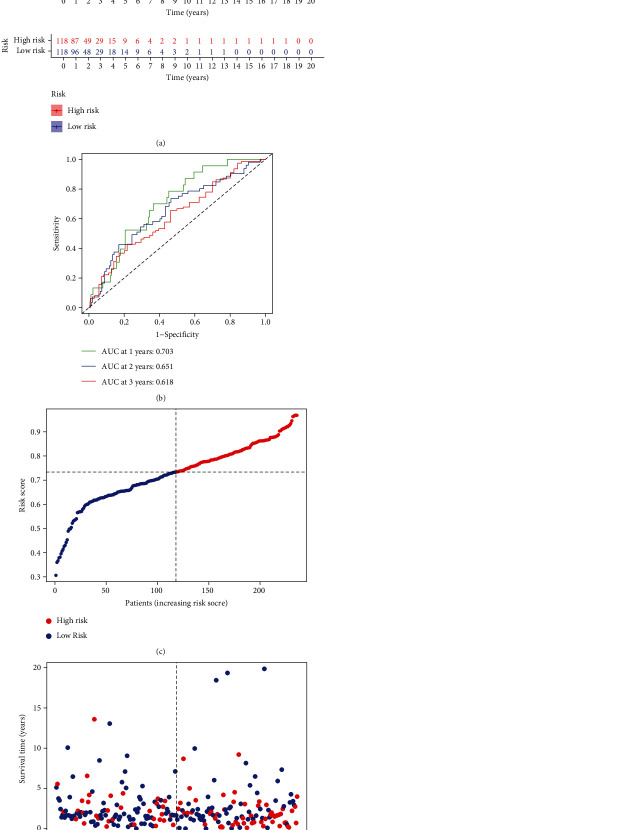
Construction of the prognostic risk models based on TCGA training cohort. (a) Survival probability in the low-risk/high-risk group. (b) Prognostic value evaluation of model using time-specific ROC curves and dynamic AUC lines. (c) Survival status of patients with LUAD (High-risk group: right of dotted line; low-risk group: left of dotted line). (d) Survival status scatters plots.

To better predict the prognosis of patients with LUAD, a prognostic model was constructed. Firstly, we screened out the PRGs associated with prognosis by univariate regression analysis, of which BAK1 and CYCS had relatively higher risks and were associated with poor survival rates (Table 1). LUAD patients were randomly divided into two cohorts according to a ratio of 5 : 5 for the training cohort (*n* = 236) and the testing cohort (n = 232). Lasso regression analysis was performed to construct a prognostic risk model based on the expression values of five PRGs associated with prognosis in the training cohort (Figure S4). The risk score was calculated as follows: RiskScore = (0.0128 × BAK1 expression level) + (0.0015 × CYCS expression level) + (−0.0660 × NLRC4 expression level) + (−0.0443 × NLRP1 expression level) + (−0.0517 × NOD1 expression lev). All LUAD patients were divided into low-risk or high-risk groups based on the median risk score calculated by the above formula (Figure 3(c)). Kaplan-Meier curves showed that patients had a shorter OS time in the high-risk group (Figure 3(a)) than in the low-risk group. This is further verified in the test queue and GEO dataset (Figure S6). In addition, the ROC curve further demonstrated that PRGs have good prediction performance in the training cohort (Figure 3(b)). An increase in the risk score was found to be associated with an increase in patient deaths (Figure 3(d)). Similar results were observed in the testing cohort (Figure S5).

### 3.4. Independent Prognostic Analysis of the Risk Model

**Figure 4:**
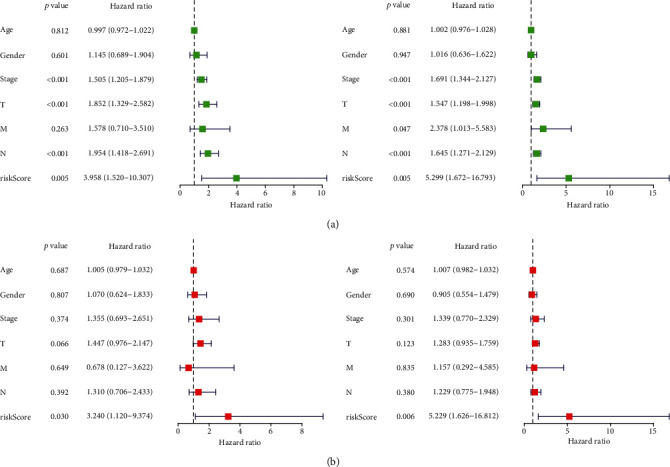
Univariate and multivariate regression analyses were performed to assess the prognostic value of risk scores and other clinical features. (a) The results of univariate regression analysis(Training Cohort: On the left side of the Figure 4(a); Testing Cohort: On the right side of the Figure 4(a)). (b) The results of multivariate regression analysis (Training Cohort: On the left side of the Figure 4(b); Testing Cohort: On the right side of the Figure 4(b)).

The clinical stage, T stage, N stage, and risk score of patients with LUAD were found to be closely correlated with OS through univariate regression analysis (Figure 4(a)). Further, risk score could be used as an independent prognostic factor for patient survival in the multivariate Cox regression analysis (Figure 4(b)). These results suggest that this model is an independent prognostic factor.

### 3.5. Relationship between Risk Model and Clinical Features, and Clustering and Risk Scores

**Figure 5:**
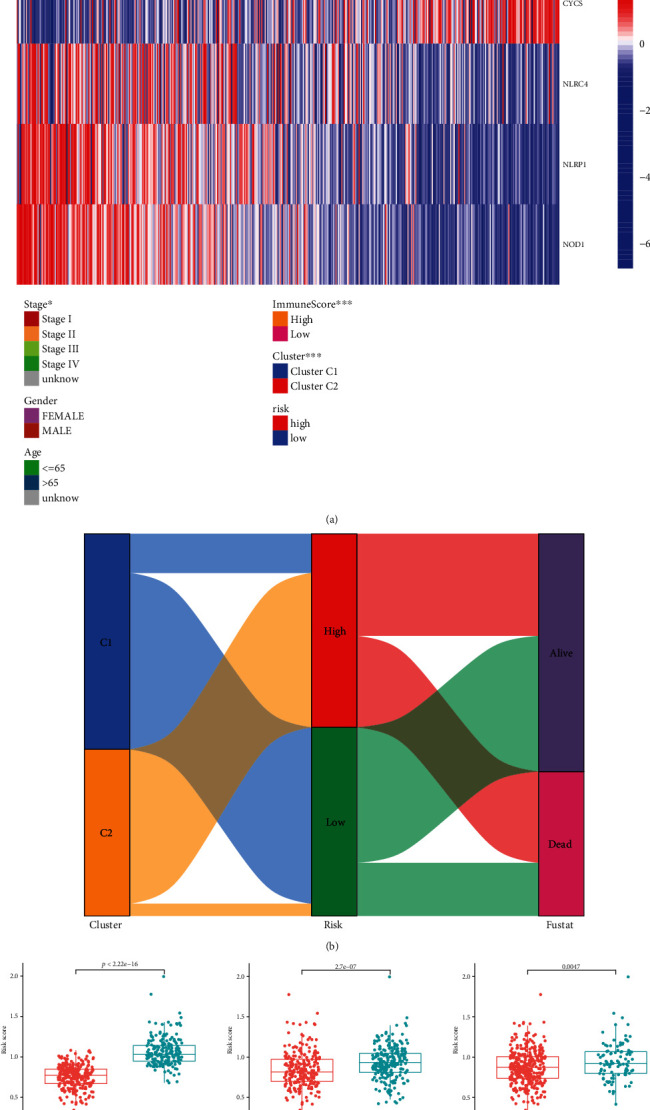
Relationship between risk model and clinical features, and clustering and risk scores. (a) The differences in immune score, subtype, and gene expression between high and low risk groups were demonstrated by heat map. (b) The Sankey diagram shows the relationship between the LUAD subtype and the high and low risk groups. (c) The differences in cluster, immune score, and clinical stage in high/low risk groups.

The heat map revealed significant differences in LUAD clustering and immunity scores between the high-risk and low-risk groups. Further, the expression levels of BAK1 and CYCS were found to be significantly increased in the high-risk group compared with those in the low-risk group. In contrast, the expression of NLRC4, NLRP1, and NOD1 was higher in the low-risk group than in the high-risk group (Figure 5(a)). More specifically, compared with those in cluster 2, the risk scores in cluster 1 were significantly lower and more patients were alive (Figure 5(b)), which is consistent with the finding that the OS of cluster 1 is greater than that of 2, thereby further verifying the reliability of clustering. Patients with high immune scores had significantly lower risk scores, and the risk score of pathological stage I-II was lower than that of III-IV (Figure 5(c)).

### 3.6. Evaluation of Tumor Immune Microenvironment and Tumor Immunotherapy Response Using Prognostic Risk Models

**Figure 6:**
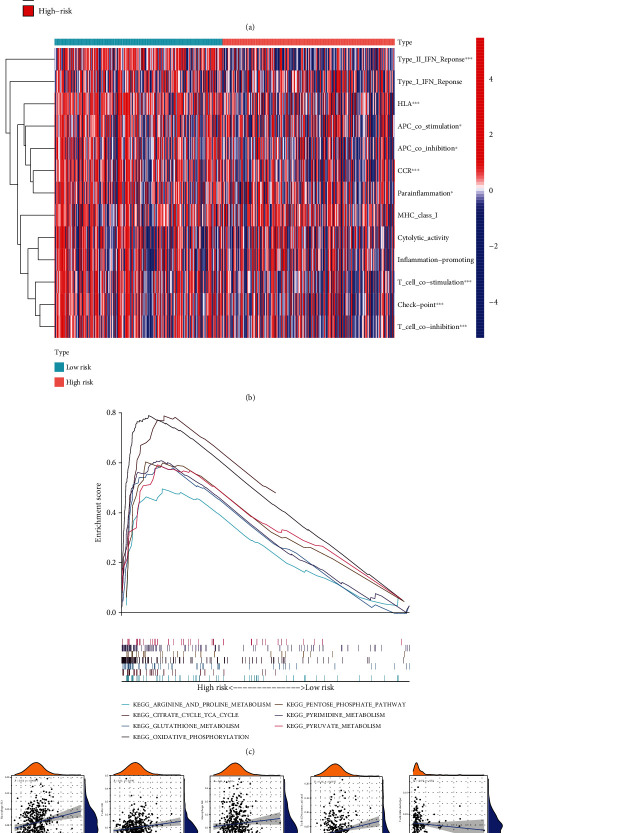
Evaluation of tumor immune microenvironment and tumor immunotherapy response using prognostic risk models. (a) The differences in TMB score and TIDE score between high- and low-risk groups. (b) Heat map of the distribution of 13 immune-related genes between the low-and high-risk groups. (c) GSEA showed high enrichment of KEGG. (d) Associations between the risk score and infiltration levels of nine immune cell types.

Immune checkpoint inhibitors were demonstrated to have different therapeutic effects on patients with LUAD in the high- and low-risk groups; the TIDE of the high-risk group was lower (*P* < 0.001). In addition, by calculating TMB, we found that it was higher in patients with a high-risk score than in those with a low-risk score (*P* = 4.8*e* − 05) (Figure 6(a)). Our results suggest that immune checkpoint blockade (ICB) treatment may be effective in patients with high-risk subtypes of LUAD. A follow-up analysis was also performed in this study. The risk score was found to have a weak positive correlation trend with the infiltration of four immune cells: M0 cells (*r* = 0.11, P < 0.02), M1 cells (*r* = 0.18, *P* = 0.00014), CD4 + T memory cells (*r* = 0.18, *P* = 0.0012), and CD8 + T cells (*r* = 0.11, *P* = 0.019) (Figure 6(d)). However, the immune response was suppressed in cluster2 (Figure 6(b)). By performing GSEA, we found that various metabolic reactions were significantly enriched in the high-risk group. KEGG signaling showed that the expression levels of arginine and proline metabolism, citrate cycle (TCA cycle), glutathione metabolism, oxidative phosphorylation, pentose phosphate pathway, pyrimidine metabolism, and pyruvate metabolism were high (Figure 6(c)).

### 3.7. Screening Potential Drugs

**Figure 7:**
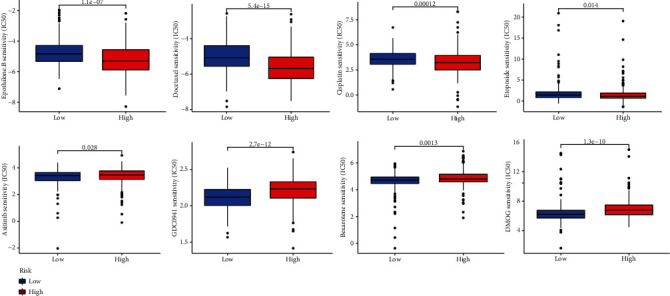
Potential therapeutic drugs for high-risk and low-risk groups.

To identify potential drugs that could be included in our pyroptosis model as treatment for LUAD patients, we selected 34 drugs closely related to LUAD.  Figure 7 shows four drugs, including axitinib, with the potential to treat low-risk patients.

## 4. Discussion

Pyroptosis is caspase-dependent cell death pathway closely associated with the immune response which may induce tumor cell lysis and the inflammatory response [25]. In contrast, many antitumor effects are also enhanced following tumor cell death and the subsequent modulation of the TME [26]. Pyroptosis stimulates the immune system influencing the TME and the efficacy of tumor immunotherapy by increasing the number of immune cells and immune regulators within the tumor niche and while immunotherapy is largely effective in LUAD there are still limitations [27]. In addition, since most studies mostly focus on the intrinsic oncogenic pathways of LUAD, it is still necessary to clarify the impact of pyroptosis on the TME and immunotherapeutic response of LUAD.

Here, we explored the expression of PRGs in LUAD, their prognostic and therapeutic value, and their impact on the TME. We analyzed the expression of 43 PRGs in LUAD and found that these genes were upregulated or downregulated in both tumor and control samples, suggesting that PRGs play an important role in the occurrence and development of LUAD. LUAD samples were then further divided into clusters 1 and 2 based on any similarities in their PRG expression profile. In addition, both clusters presented with different prognostic and clinical features, with cluster 2 presenting with significantly different immune scores and immune cell infiltration patterns from cluster 1, suggesting distinct differences in their TMEs. These differences in microenvironment may lead to differences in OS and PFS. These evaluations also revealed an increase in the proportion of quiescent dendritic cells, quiescent mast cells, monocytes, and quiescent CD4+ T memory cells in cluster1 and an increase in follicular helper T cells in cluster2. Tamminga et al. and Qiu et al. suggested that this suggests that the normal function of the follicular helper T cell subpopulation, as described in NSCLC, may be impaired in these tissues resulting in decreased differentiation of specific B cells, indirectly impairing the humoral immune response and promoting tumor growth [28, 29]. It is also worth noting that LUAD patients with higher immune scores exhibited a higher survival rate than those with lower immune score [30]. Therefore, the survival time for cluster2 patients was shorter than that of cluster1 patients. Thus, we concluded that pyroptosis may play an important role in determining survival and establishing the TME in LUAD patients.

Given this, we went on to evaluate the prognostic value of five PRGs, BAK1, CYCS, NLRC4, NLRP1, and NOD1 in LUAD patients. BAK1 is a member of the Bcl-2 family known to interact with the mitochondria to induce apoptosis [31]. BAK1 is transferred to the mitochondria after receiving a stimulatory signal that induces apoptosis where it induces pore creation in the outer mitochondrial membrane (MOM) through oligomerization increasing permeability and dysregulation of the electron transport chain [32]. This induces the release of various apoptotic factors, including cytochrome c, into the cytoplasm where they activate caspase-9, which is then processed and activated by the caspase-3 [33]. Activated caspase-3 cleaves gasdermin-E (GSDME) to form GSDME-NT fragments, resulting in cell membrane perforation and inducing pyroptosis [34]. Notably, we also found that BAK1 expression was closely associated with a poor prognosis in LUAD. The CYCS gene encodes cytochrome c, which is closely related to the synthesis of ATP and the survival of tumor cells [35]. The binding of the apoptotic factor CYCS to Apaf-1 triggers the activation of caspase-9, which then activates caspase-3 which cleaves GSDME activating pyroptosis [36]. Our results suggest that CYCS is highly expressed in LUAD tissues and is associated with poor prognosis in LUAD patients. This is supported by Jamsheed et al. who found that serum cytochrome c levels in patients with LUAD were more likely to experience severe disease and present with a poor prognosis [37]. This inflammasome is a multiprotein complex consisting of an N-terminal caspase recruitment domain (CARD), an intermediate nucleotide binding oligomerization domain (NOD), and a leucine-rich repeat (LRR) that cleaves procaspase-1, promoting the cleavage and secretion of mature IL-1*β* and IL-18, inducing pyroptosis [38]. NLRC4 is less well established in tumors. However, Tenthorey et al. found that NLRC4 and caspase-1 are involved in tumor progression and promote breast and colon cancer metastasis in obese mice [39]. Despite this, evaluations of NLRC4 in LUAD are still lacking. Our study found that high expression levels of NLRC4 in the low-risk group were associated with better prognosis, but the specific mechanism underlying this effect requires further evaluation. NLRP1 regulates innate and adaptive immune responses via the inflammasome and Shen et al. also found that NLRP1 may improve the prognosis of patients with LUAD, which is consistent with our findings. Since the expression of NLRP1 is positively correlated with the infiltration of tumor immune cells, this may provide clues into its underlying mechanism [40]. NOD1, is a member of the nod-like receptor (NLR) family and recognizes pathogenic microorganisms often facilitating the activation of the innate immune system [41]. Its activation promotes the maturation and secretion of IL-1*β* and IL-18, cleavage of GSDMD, and ultimately leads to pyroptosis [42]. In addition, our data suggests that NOD1 may act as a tumor suppressor gene (HR< 1) in LUAD. Similarly, Liu et al. found that NOD1 knockout mice were more sensitive to inflammatory colon tumors than nonknockout mice [43].

Based on the prognostic risk model constructed using these five PRGs, patients with LUAD were divided into different risk groups. The OS of patients in the high-risk group was significantly lower than that of patients in the low-risk group, and where primarily distributed within cluster 2, presenting with a lower immune score. We then used this model to predict the sensitivity of different risk groups to immunotherapy by evaluating changes in their TMB and TIDE expression levels. The high-risk group had a significantly higher TMB than the low-risk group, supporting our hypothesis of better immune response to ICIs. The TIDE algorithm is widely used in LUAD research and Jiang et al. demonstrated that tumor immune escape can be simulated using the TIDE algorithm, which integrates features of T cell dysfunction and rejection, and can predict the clinical response of tumor patients to ICB based on pretreatment tumor conditions [44]. This means that the lower the TIDE algorithm, the better the patient's response to immunotherapy. When we combined the TIDE and TMB expression levels, we found that the high-risk group was likely to respond better to immunotherapy than the low-risk group, which suggests that our prognostic model may also serve to predict immunotherapy response, helping to personalize LUAD treatment.

This prompted us to further explore the reasons for these differences in susceptibility. These assessments showed a trend toward increased infiltration of M0 macrophages, M1 macrophages, CD4+ T memory, and CD8+ T cells in the high-risk group. Subsequent immune pathway analysis revealed that this facilitated an increase in the inhibition of many immune pathways in these high-risk populations. In addition, these evaluations revealed that there were also a significant number of metabolism-related pathways that were highly expressed in the high-risk group suggesting that while the high degree of immune infiltration was likely to prevent tumor growth extensive metabolic reprogramming may allow these tissue to circumvent these protective mechanisms [45]. Tumor metabolic reprogramming plays an important role in promoting tumor growth, proliferation, invasion, metastasis, and immunosuppression [46, 47]. Therefore, we suggest that the combined use of metabolic pathway inhibitors and immunotherapy may be beneficial for these patients. It is worth noting that, we also went on to screen a series of potential drugs, including Axitinib, Bexarotene, and DMOG, for patients who are less sensitive to immunotherapy.

Pyroptosis-related genes are rarely studied in LUAD patients and have certain research significance. This study innovatively used a consistent clustering method to subclassify LUAD and explored the mechanisms underlying the differences in patient survival from an immunological perspective. Our study provided a new approach to explain tumor heterogeneity. Furthermore, we constructed a prognostic model for clinical application. In addition, immunotherapy-sensitive LUAD patients were screened according to TMB and more consistent and actual conditions and scientific TIDE scores, providing potential drug choices for patients who were less sensitive to immunotherapy. However, our study has some limitations, and further experiments are needed to verify the biological significance of PRGs in LUAD.

## 5. Conclusions

In summary, our study demonstrates that pyroptosis affects the outcome of LUAD and the efficacy of immunotherapy by acting on the TME. Using five PRGs to construct a prognostic risk model we showed that pyroptosis is an independent risk factor; this finding provides a new realistic approach for evaluating patients' prognosis, predicting their sensitivity to immunotherapy, and exploring potential therapeutic agents.

## Figures and Tables

**Table 1:** 5 prognostic genes associated with pyroptosis.

GENE	HR	HR.95 L	HR.95H	*P* value
BAK1	1.027687	1.007991	1.047769	0.005672
CYCS	1.011209	1.002994	1.019492	0.007396
NLRC4	0.759973	0.621575	0.929186	0.007449
NLRP1	0.892924	0.814924	0.978389	0.015164
NOD1	0.911358	0.85142	0.975515	0.007491

## Data Availability

The data for this study are publicly available and can be freely downloaded from TCGA database and GEO database.

## Supplementary Materials

Supplementary Materials Figure S1: when *K* = 2, the cluster has good stability (*K* = 1 − 9). Figure S2: through PCA verification, cluster1/cluster2 can be well-divided into two categories. Figure S3: relationship between different subtypes of LUAD and immune cell infiltration. Figure S4: Lasso Regression analysis for TCGA Training cohort. Figure S5: further validation is performed in the validation group. Figure S6: differences in survival between high and low risk based on GEO database. Table S1: expression of pyroptosis related genes in LUAD. Table S2: ESTIMATE Score, Immune Score, and Stromal Score of patients with LUAD. Table S3: the proportion of 22 immune cell types in LUAD. Table S4: RiskScore. Table S5: differential expression of pyroptosis related genes in LUAD and normal samples.
