# Supplementary material for: Subtype Classification, Immune Infiltration, and Prognosis Analysis of Lung Adenocarcinoma Based on Pyroptosis-Related Genes
Source: Biomed Res Int. 2022 Oct 12;2022:1371315. doi: 10.1155/2022/1371315 (PMC9581708; doi:10.1155/2022/1371315)
Supplement: Supplementary Materials — Figure S1: when K = 2, the cluster has good stability (K = 1 − 9). Figure S2: through PCA verification, cluster1/cluster2 can be well-divided into two categories. Figure S3: relationship between different subtypes of LUAD and immune cell infiltration. Figure S4: Lasso Regression analysis for TCGA Training cohort. Figure S5: further validation is performed in the validation group. Figure S6: differences in survival between high and low risk based on GEO database. Table S1: expression of pyroptosis related genes in LUAD. Table S2: ESTIMATE Score, Immune Score, and Stromal Score of patients with LUAD. Table S3: the proportion of 22 immune cell types in LUAD. Table S4: RiskScore. Table S5: differential expression of pyroptosis related genes in LUAD and normal samples. [file 1371315.f1.zip › Figure S1.pdf]

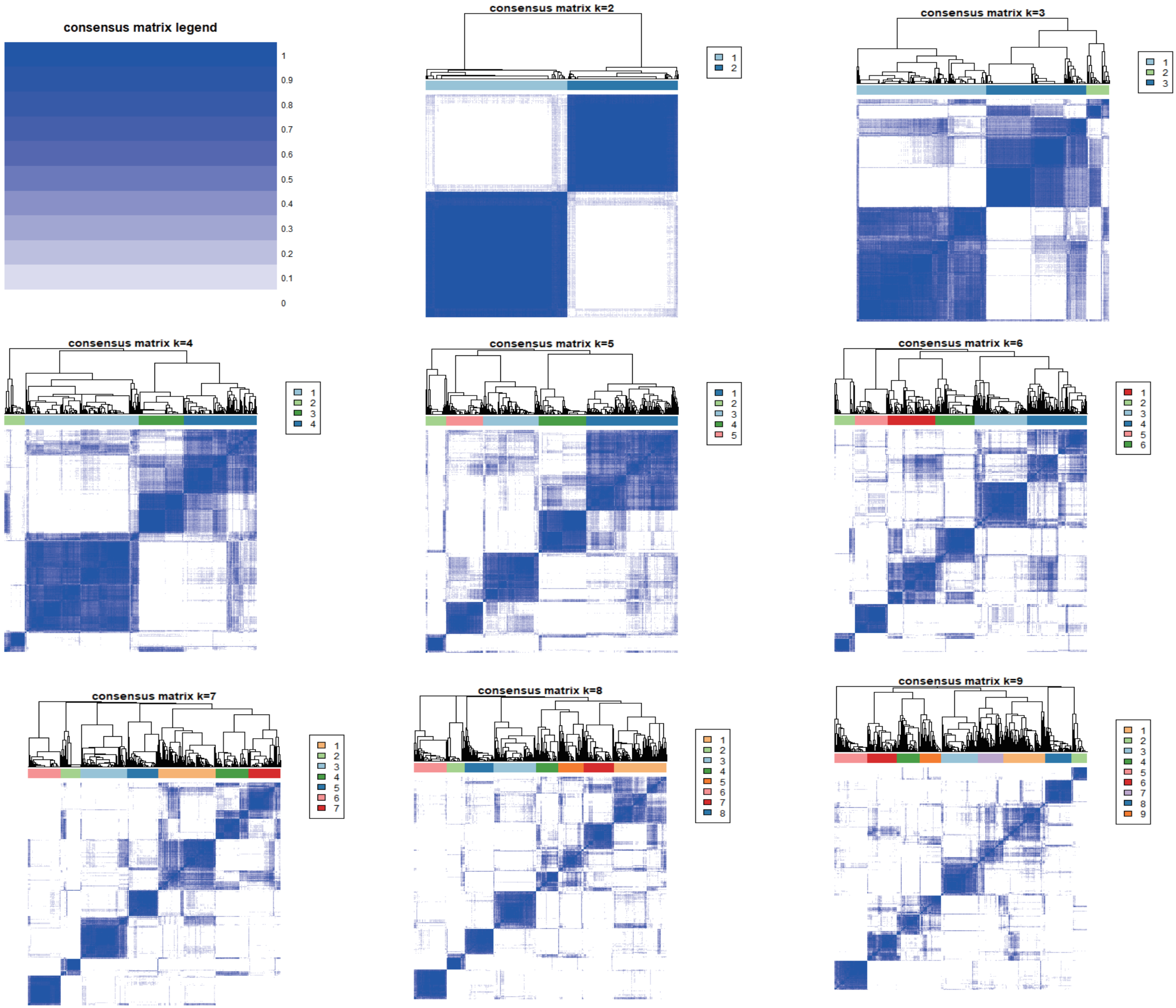
consensus matrix legend
1
0.9
0.8
0.7
0.6
0.5
0.4
0.3
0.2
0.1
0
consensus matrix k=2
1
2
consensus matrix k=3
1
2
3
consensus matrix k=4
1
2
3
4
consensus matrix k=5
1
2
3
4
5
consensus matrix k=6
1
2
3
4
5
6
consensus matrix k=7
1
2
3
4
5
6
7
consensus matrix k=8
1
2
3
4
5
6
7
8
consensus matrix k=9
1
2
3
4
5
6
7
8
9
