# Supplementary figures and images for: Subtype Classification, Immune Infiltration, and Prognosis Analysis of Lung Adenocarcinoma Based on Pyroptosis-Related Genes
Source: Biomed Res Int. 2022 Oct 12;2022:1371315. doi: 10.1155/2022/1371315 (PMC9581708; doi:10.1155/2022/1371315)

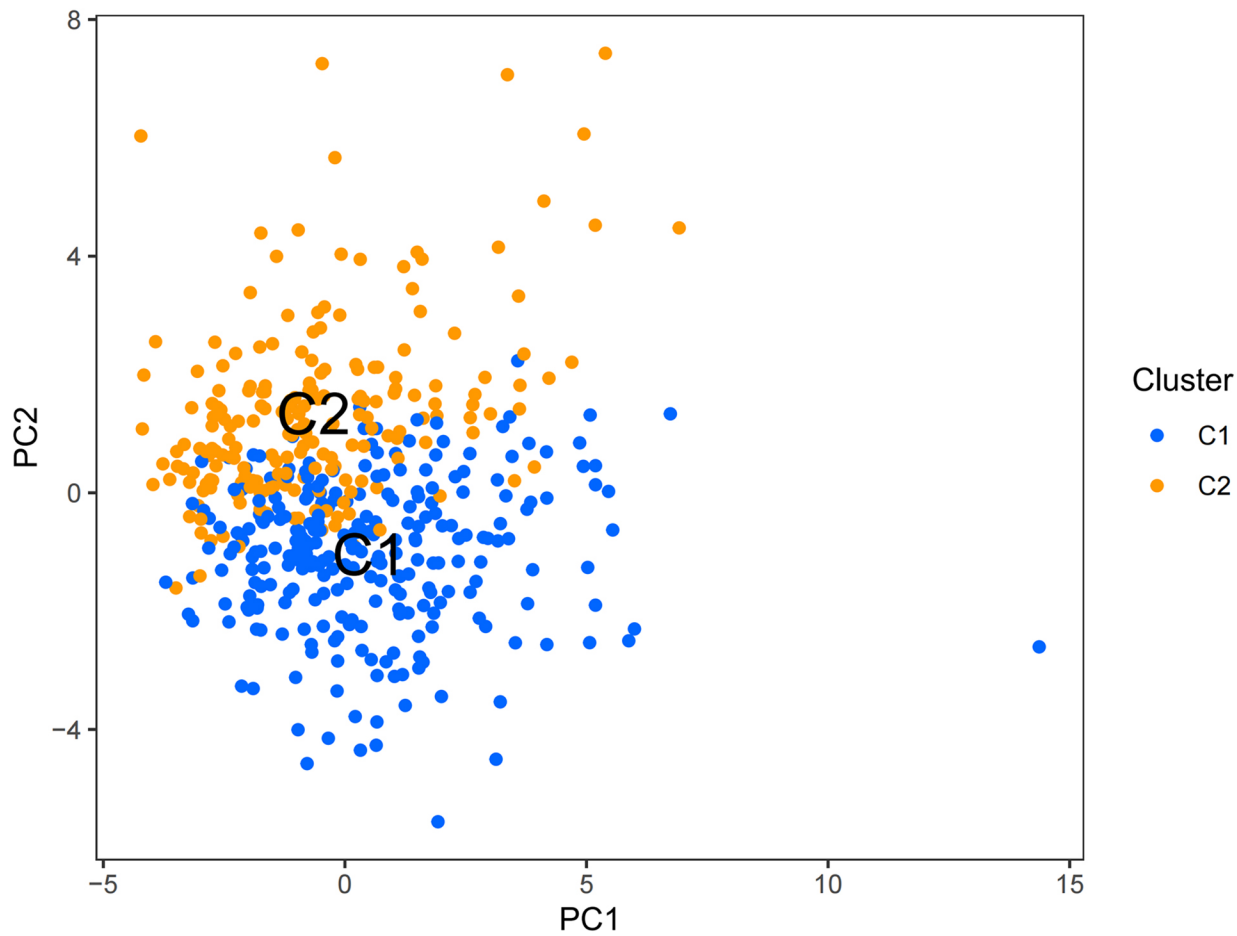

Supplement: Supplementary Materials — Figure S1: when K = 2, the cluster has good stability (K = 1 − 9). Figure S2: through PCA verification, cluster1/cluster2 can be well-divided into two categories. Figure S3: relationship between different subtypes of LUAD and immune cell infiltration. Figure S4: Lasso Regression analysis for TCGA Training cohort. Figure S5: further validation is performed in the validation group. Figure S6: differences in survival between high and low risk based on GEO database. Table S1: expression of pyroptosis related genes in LUAD. Table S2: ESTIMATE Score, Immune Score, and Stromal Score of patients with LUAD. Table S3: the proportion of 22 immune cell types in LUAD. Table S4: RiskScore. Table S5: differential expression of pyroptosis related genes in LUAD and normal samples. [file 1371315.f1.zip › Figure S2.pdf]

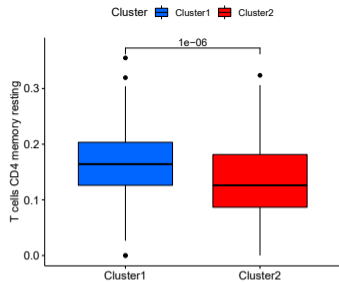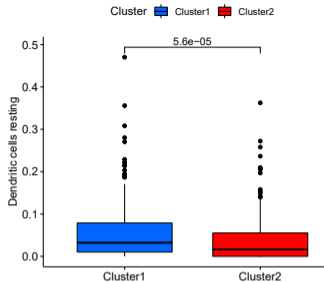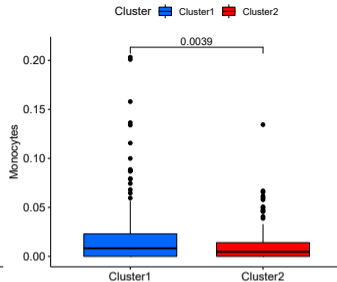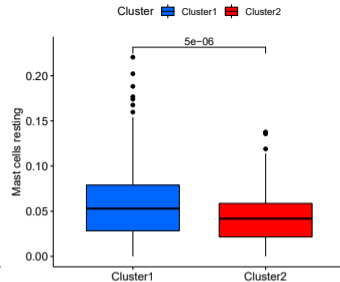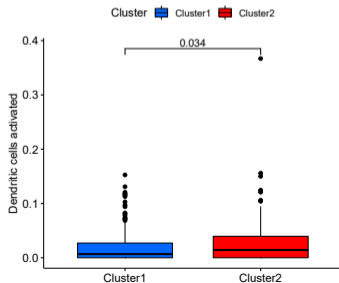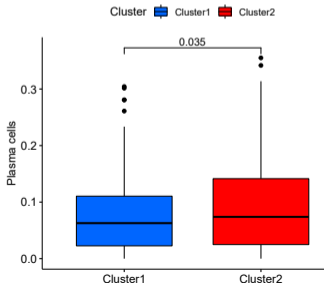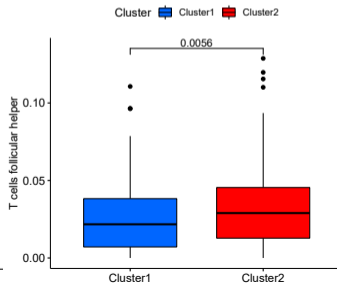

Supplement: Supplementary Materials — Figure S1: when K = 2, the cluster has good stability (K = 1 − 9). Figure S2: through PCA verification, cluster1/cluster2 can be well-divided into two categories. Figure S3: relationship between different subtypes of LUAD and immune cell infiltration. Figure S4: Lasso Regression analysis for TCGA Training cohort. Figure S5: further validation is performed in the validation group. Figure S6: differences in survival between high and low risk based on GEO database. Table S1: expression of pyroptosis related genes in LUAD. Table S2: ESTIMATE Score, Immune Score, and Stromal Score of patients with LUAD. Table S3: the proportion of 22 immune cell types in LUAD. Table S4: RiskScore. Table S5: differential expression of pyroptosis related genes in LUAD and normal samples. [file 1371315.f1.zip › Figure S3.pdf]

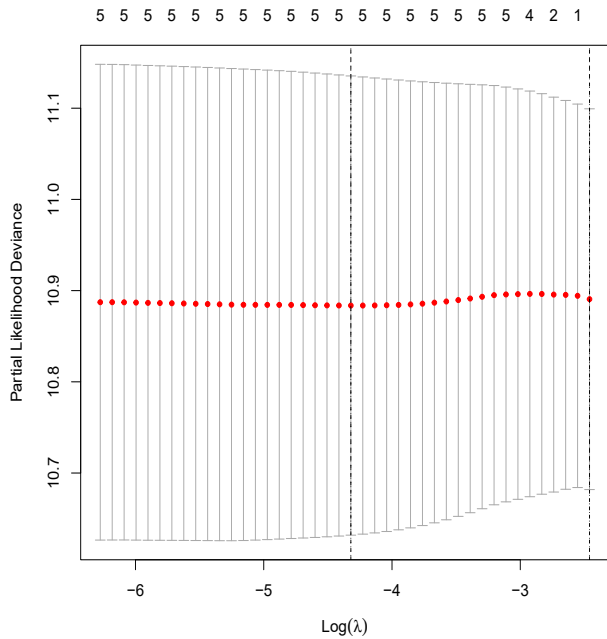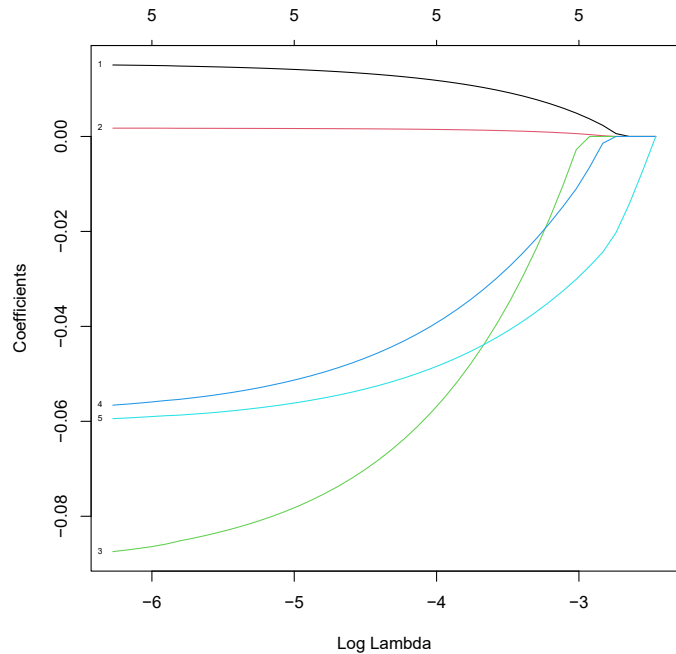

Supplement: Supplementary Materials — Figure S1: when K = 2, the cluster has good stability (K = 1 − 9). Figure S2: through PCA verification, cluster1/cluster2 can be well-divided into two categories. Figure S3: relationship between different subtypes of LUAD and immune cell infiltration. Figure S4: Lasso Regression analysis for TCGA Training cohort. Figure S5: further validation is performed in the validation group. Figure S6: differences in survival between high and low risk based on GEO database. Table S1: expression of pyroptosis related genes in LUAD. Table S2: ESTIMATE Score, Immune Score, and Stromal Score of patients with LUAD. Table S3: the proportion of 22 immune cell types in LUAD. Table S4: RiskScore. Table S5: differential expression of pyroptosis related genes in LUAD and normal samples. [file 1371315.f1.zip › Figure S4.pdf]

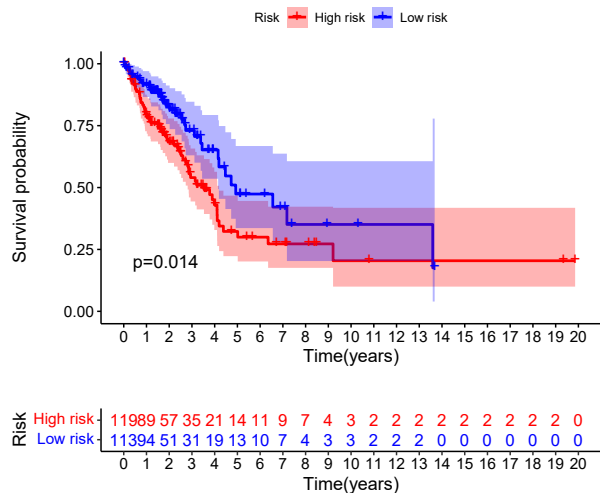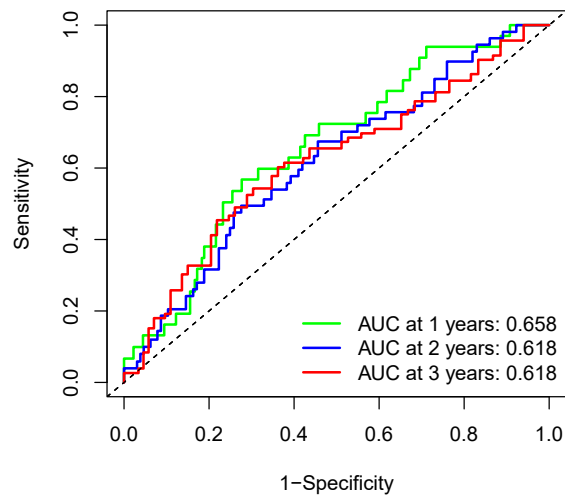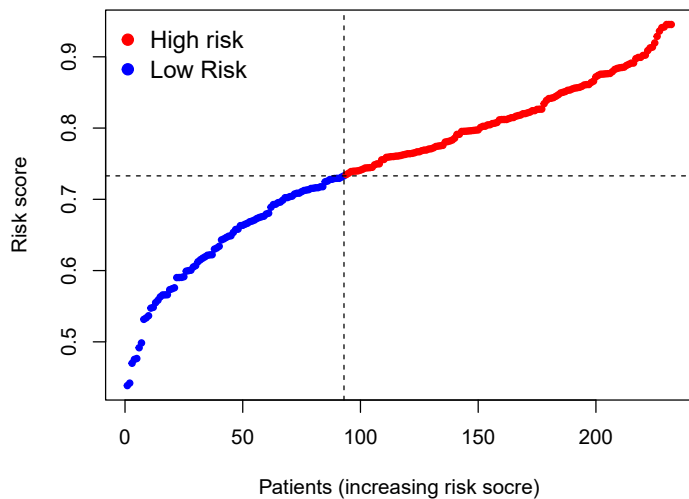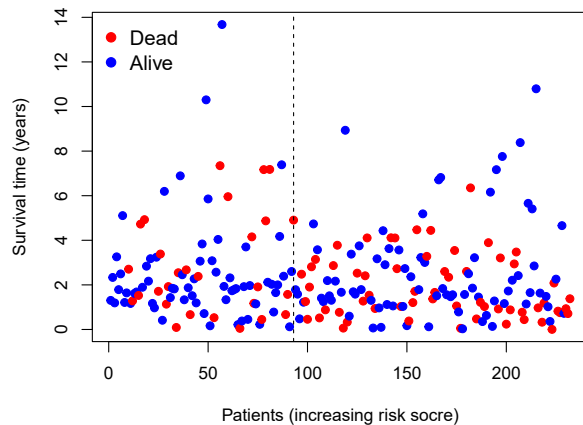

Supplement: Supplementary Materials — Figure S1: when K = 2, the cluster has good stability (K = 1 − 9). Figure S2: through PCA verification, cluster1/cluster2 can be well-divided into two categories. Figure S3: relationship between different subtypes of LUAD and immune cell infiltration. Figure S4: Lasso Regression analysis for TCGA Training cohort. Figure S5: further validation is performed in the validation group. Figure S6: differences in survival between high and low risk based on GEO database. Table S1: expression of pyroptosis related genes in LUAD. Table S2: ESTIMATE Score, Immune Score, and Stromal Score of patients with LUAD. Table S3: the proportion of 22 immune cell types in LUAD. Table S4: RiskScore. Table S5: differential expression of pyroptosis related genes in LUAD and normal samples. [file 1371315.f1.zip › Figure S5.pdf]

Risk High risk Low risk

Survival probability

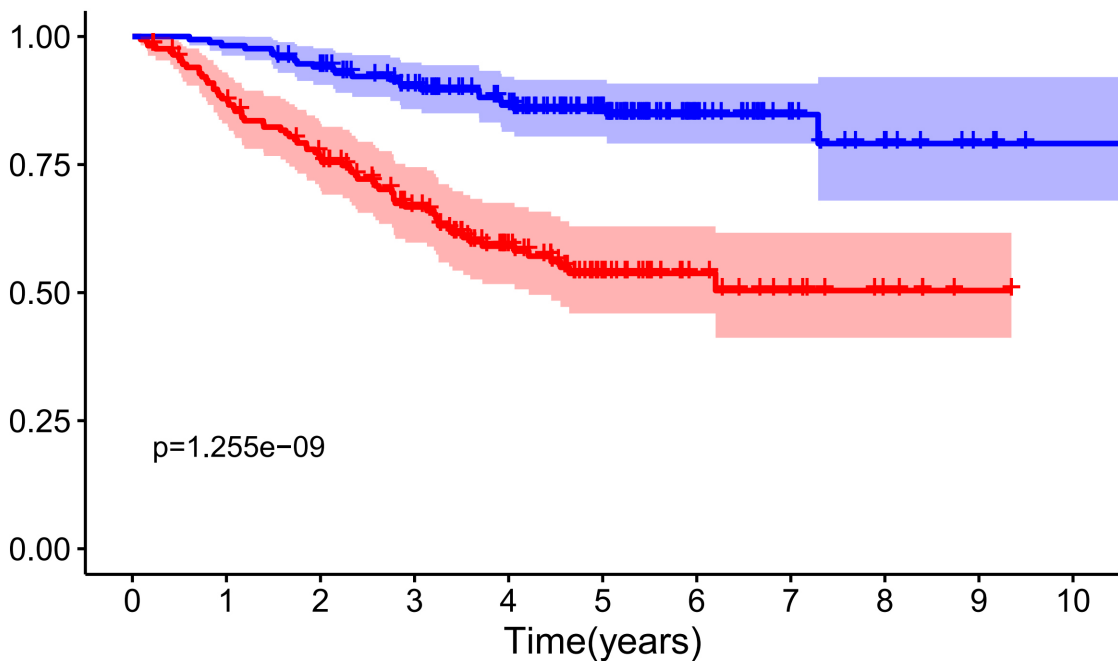

$p=1.255e-09$

Risk High risk Low risk

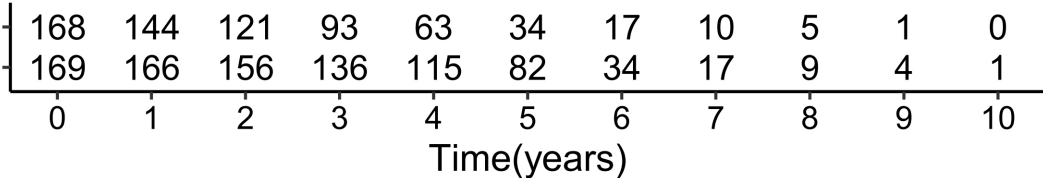

Supplement: Supplementary Materials — Figure S1: when K = 2, the cluster has good stability (K = 1 − 9). Figure S2: through PCA verification, cluster1/cluster2 can be well-divided into two categories. Figure S3: relationship between different subtypes of LUAD and immune cell infiltration. Figure S4: Lasso Regression analysis for TCGA Training cohort. Figure S5: further validation is performed in the validation group. Figure S6: differences in survival between high and low risk based on GEO database. Table S1: expression of pyroptosis related genes in LUAD. Table S2: ESTIMATE Score, Immune Score, and Stromal Score of patients with LUAD. Table S3: the proportion of 22 immune cell types in LUAD. Table S4: RiskScore. Table S5: differential expression of pyroptosis related genes in LUAD and normal samples. [file 1371315.f1.zip › Figure S6.pdf]
